# Knowledge-enhanced prototypical network with class cluster loss for few-shot relation classification

**DOI:** 10.1371/journal.pone.0286915

**Published:** 2023-06-08

**Authors:** Tao Liu, Zunwang Ke, Yanbing Li, Wushour Silamu

**Affiliations:** 1 College of Software, Xinjiang University, Urumqi, China; 2 Xinjiang Multilingual Information Technology Laboratory, Xinjiang University, Urumqi, China; 3 Xinjiang Multilingual Information Technology Research Center, Xinjiang University, Urumqi, China; Anhui University, CANADA

## Abstract

Few-shot Relation Classification identifies the relation between target entity pairs in unstructured natural language texts by training on a small number of labeled samples. Recent prototype network-based studies have focused on enhancing the prototype representation capability of models by incorporating external knowledge. However, the majority of these works constrain the representation of class prototypes implicitly through complex network structures, such as multi-attention mechanisms, graph neural networks, and contrastive learning, which constrict the model’s ability to generalize. In addition, most models with triplet loss disregard intra-class compactness during model training, thereby limiting the model’s ability to handle outlier samples with low semantic similarity. Therefore, this paper proposes a non-weighted prototype enhancement module that uses the feature-level similarity between prototypes and relation information as a gate to filter and complete features. Meanwhile, we design a class cluster loss that samples difficult positive and negative samples and explicitly constrains both intra-class compactness and inter-class separability to learn a metric space with high discriminability. Extensive experiments were done on the publicly available dataset FewRel 1.0 and 2.0, and the results show the effectiveness of the proposed model.

## 1 Introduction

Relation classification (RC) [1] is a basic information extraction mission that tries to comprehend natural language text and determine the relation between entity pairs. For example, "Sofia is the capital city of Bulgaria." represents the relation "capital of" between "Sofia" and "Bulgaria."

Traditional relation classification methods are primarily trained using fully supervised data [2], and their model performance is dependent on the amount of labeled data available. However, manual labeling is an expensive, labor-intensive, and time-consuming task, making it challenging to generalize fully supervised models to specific domains. To reduce the expense of labeling work, Mintz et al. [3] developed a distant supervised method to automatically label training samples from a corpus by entity alignment. Unfortunately, the labeling strategy is too absolute: instances containing two identical entities do not necessarily express the same relation as in the knowledge base, which results in some noise in the dataset construction. In addition, due to the uneven distribution of samples in the corpus, many relations under the long-tail distribution still lack sufficient training data, resulting in a significant decline in model performance. Therefore, it is necessary to research the relation classification task with insufficient training data.

In fact, people only need a few instances to quickly acquire anything new. Even a young child of just a few years can tell what makes each animal unique by looking at a few cartoon pictures of them. This sparked the idea for few-shot learning (FSL) [4], which tries to learn and handle problems with a few labeled data. Early FSL research was primarily focused on computer vision [5]. Recently, FSL has gradually expanded into natural language processing. Because of the diversity and complexity of natural language, the few-shot text classification task is more challenging than the classification of images.

Few-shot Relation Classification (FSRC) focuses on learning the generic knowledge that is built into different relation classes through multi-task learning. This allows the model to quickly process new tasks with a few instances so that it can generalize to real-world application scenarios. Table 1 shows a task example of FSRC. The learning objective of FSRC is to accurately predict the query instance’s relation.

**Table 1:** An example of 5-way-1-shot task setting of FSRC.

Training Data	Relation	Instance
**Support Set**	R1: follower of	The **DeSoto Bridge** across the *Mississippi River* in St.
	R2: fare zone	**Herm** is one of the *Channel Islands* in the English Channel.
	R3: child	Mukesh is married to **Nita Ambani** and has two sons, Anant and *Akash*.
	R4: member of	South Africa is part of the *IBSA Dialogue Forum*, alongside **Brazil** and India.
	R5: solves	**NGC 285** is a lenticular galaxy in the constellation *Cetus*.
**Query Set**	R1, R2, R3, R4 or R5	In his first game, he had played alongside the brothers Tom and **Ken Graveney**; in his last game, he played alongside Ken ’s son *David*.

The text in bold and italics represents head and tail entities, respectively. Each instance’s relation labels are displayed on the left side of the table. The goal of the model is to accurately identify the relation R3 from the query instance.

Recent years have seen a gradual increase in FSRC research. Sun et al. [6] propose a hierarchical attention prototypical network that restricts the model to the significance of each word in a given instance for relational classification. Ye et al. [7] propose a multi-level matching aggregation network to improve the class prototype. To provide the model with reliable prediction indicators, Yang et al. [8] introduce text descriptions for prototype representation. Han et al. [9] propose a pre-training method that introduces relation labels and relation description information from a large dataset during the pre-training phase to improve sentence representation.

However, the majority of existing methods constrain the generation of class prototypes through complex network structures, which introduce numerous meaningless or even harmful parameters and typically have low bias and high variance [10]. In addition, due to the random sampling strategy for training, the mode with triplet loss [11] is prone to local optimization and erroneous decisions for these challenging few-shot tasks with high semantic similarity. Even though Xiao et al. [12] introduce hard sample mining to triplet loss, it still only considers the relative distance between positive and negative samples [13], which limits the model’s ability to handle outlier samples with low semantic similarity.

In this paper, a knowledge-enhanced prototypical network (KEPN) is proposed as a way to deal with the problems listed above. KEPN is a model that focuses on improving the model’s ability to represent prototypes and handle difficult classification tasks. Specifically, we design a non-weighted prototype enhancement module to explicitly filter redundant information between basic prototypes and relation information and to perform feature selection and complementation between them. Through relation label and description knowledge, the prototype enhancement module adds support information to the prototype representation and reduces the risk of overfitting. In the meantime, we designed a new loss function that focuses on intra-class compactness to help handle outlier samples better.

This paper’s contributions are summarized as follows:

We propose a non-weighted prototype enhancement method to filter and complement the features of prototypes and relation information to improve prototype representation.

We design a class cluster loss function that uses hard positive and negative samples as optimization instances and explicitly constrains both intra-class compactness and inter-class separability to discover a more discriminatory metric space.

Our model achieves competitive performance compared with other baseline models on the FSRC dataset FewRel 1.0, and ablation research and visualization were designed to demonstrate the validity of our model.

## 2 Related work

### 2.1 Few-shot learning

FSL aims to train a model that could rapidly adapt to the new assignment with a few data, which consisted of optimization-based and metric-based methods. The optimization-based method focuses to discover the optimal initialization parameters of the target task to achieve the best prediction performance in all subsequent tasks with the fewest gradient descent steps possible. MAML [14] is a model-agnostic parameter optimization algorithm that iteratively learns how to update network parameters using second-order gradients. Reptile [15] is a first-order optimization algorithm that optimizes the gradient inner product of small batches to train the initialization parameters. MAML and Reptile both focus on enhancing the overall learning capacity of the model rather than solving a specific problem. Numerous researchers have proposed a series of improvements based on this foundation. Ravi et al. [16] designed the LSTM-based model that simultaneously discovers initialization parameters and optimization rules. Dong et al. [17] propose a parameter optimization algorithm guided by meta-information. Qu et al. [18] propose a Bayesian method to generalize the various prototypes more effectively. The metric-based method is more laconic and efficient than the optimization-based method, which predicts results based on the distance between query and support samples. Siamese network [19] obtained the input sample feature vectors from two networks with shared weights and calculated the similarity using Euclidean distance. Matching networks [20] extracted the feature vectors from convolutional neural networks and analyzed distance by cosine similarity. Prototypical network [21] represents the prototype by aggregating the supported instances and classifying relations by computing the distance between query instances and each prototype. In recent years, few-shot learning has been applied in various fields. Zhang et al. [22] propose a soft distribution-aware few-shot learning strategy to segment tumors from magnetic resonance imaging data in low-resource scenarios. Feng et al. [23] propose a class-adaptive framework based on MAML to address few-shot anomaly detection in encrypted traffic. Mozafari et al. [24] attempted to combine MAML with a prototypical network and successfully applied it to the few-shot cross-lingual hate speech detection task. This paper is also based on the prototypical network, which reflects a simpler inductive bias.

### 2.2 Prototypical network

Currently, metric-based models for FSRC tasks are primarily concerned with prototypical networks. To mitigate the effects of noisy data, Gao et al. [25] proposed a hybrid network comprised of instance-level and feature-level attention. Yang et al. [26] proposed a method for enhancing entity concepts that combines the information of concepts and sentences at the word level to provide effective relation classification cues. Dong et al. [27] modeled a generic relational network via semantic mapping. Meanwhile, extensive research has been conducted on prototype networks in various other fields as well. Liu et al. [28] proposed an interaction graph-based prototypical network to solve the problem of domain transfer. Yarats et al. [29] introduce reinforcement learning to prototypical network to learn an efficient representation. However, most of these models used complex parameter networks to implicitly constrain the representation of class prototypes, thereby limiting the model’s ability to generalize to new tasks. We believe that representing class prototypes with fewer parameters and more explicit ways is advantageous. Therefore, we explore a non-weighted prototype enhancement module that explicitly filters and fuses class prototypes and relational information to enhance the generalizability of the model.

### 2.3 Triplet loss

In the metric space, the distance between prototypes with similar semantics is usually very close. This makes it hard for the model to classify the prototypes. Because of this, some works have used triplet loss to constrain the distance between the samples of different classes. Fan et al. [11] implement triplet loss to constrain the margin distances between different prototypes, allowing the model to learn a metric space with high discriminability. The effectiveness of triplet loss, however, is heavily dependent on the sampling strategy. The random sampling strategy causes the model to disregard samples with high loss, resulting in sluggish convergence in the later stages of the model and an increased propensity for local optima. Xiao et al. [12] presented hard sample mining based on triplet loss, which selects the most distant positive sample and the closest negative sample as optimization instances. It is advantageous for the model to accommodate relations with a high degree of semantic similarity. Unfortunately, only the relative distance was considered. The model remains limited in its ability to deal with outlier samples. This paper will investigate how to constrain intra-class compactness in order to learn a metric space with high discriminability.

## 3 Task definition

The majority of the FSRC is currently trained using meta-learning [30], which consists of two phases: *M*_*train*_ and *M*_*test*_. In the *M*_*train*_ phase, *K* instances are selected at random from each of the *N* classes of the dataset as a task, and multiple tasks are combined to form a support set to train the model. Additionally, *N* instance is selected from the remaining samples of the *N* classes as query set for validation, which is commonly referred to as *N*−*way*−*K*−*shot*. *M*_*test*_ contains the same task configuration as *M*_*train*_. However, the model learns the target domain data during the *M*_*test*_ phase, which does not overlap with the *M*_*train*_ class.

Consequently, FSRC can be defined as the task of learning prototype representation from a given support set $S=\{s_{k}^{n},n=1,\ldots ,N,k=1,\ldots ,K\}$ and predicting the relation *y*^*n*^ corresponding to the query instance *q*^*n*^ in the query set *Q* ={*q*^*n*^, *n* = 1,…,*N*}.

## 4 Methodology

**Fig 1:**
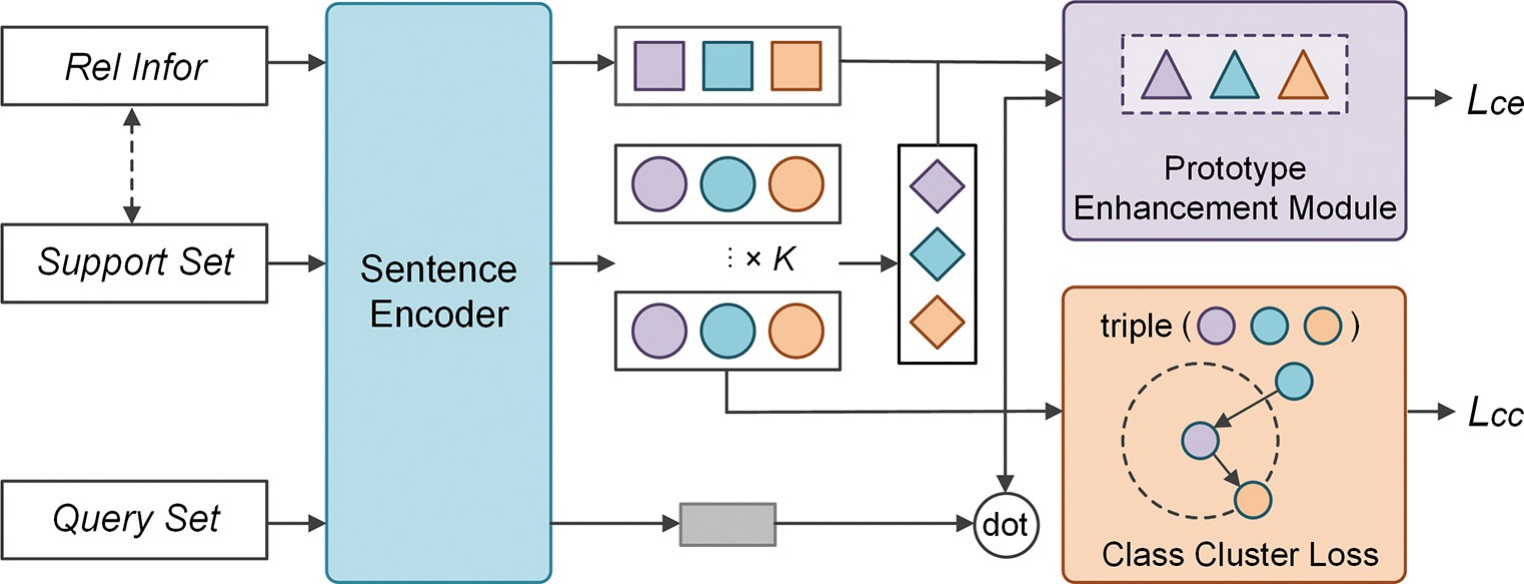
The general framework of KEPN.

In this section, we describe KEPN with class cluster loss in greater depth. The input to KEPN consists of multiple tasks sampled from a dataset, each containing a support and a query set, as depicted in Fig 1. In the meantime, relation labels and relation descriptions will also be transmitted to the encoder. All inputs are encoded using a sentence encoder to generate prototypes and relation information vectors. These vectors will be sent to the prototype enhancement module in order to enhance the prototype’s representation. Finally, a class cluster loss function is introduced to encourage the model to learn a metric space with high discriminability.

### 4.1 Sentence encoder

We use one BERT for embedding support and query instances in this work. Following the work of MTB [31], instance sentences are generated by concatenating the start tokens of the two target entities. The support and query instances are denoted as $\left\{S_{k}^{n}\in R^{2d},n=1,\ldots ,N,k=1,\ldots ,K\right\}$ and {*Q*^*n*^∈*R*^2*d*^, *n* = 1,…,*N*} respectively, and the hidden layer size of the sentence encoder is denoted as *d*. We concatenate the relation label and description information for each relation class and input them into the sentence encoder to obtain two relation feature vectors. In other words, the sentence feature vector corresponding to the "[CLS]" token represented the global representation of the relation *R*_*glo*_, and the mean of all word feature vectors represented the local representation of the relation *R*_*loc*_.

### 4.2 Prototype enhancement module

**Fig 2:**
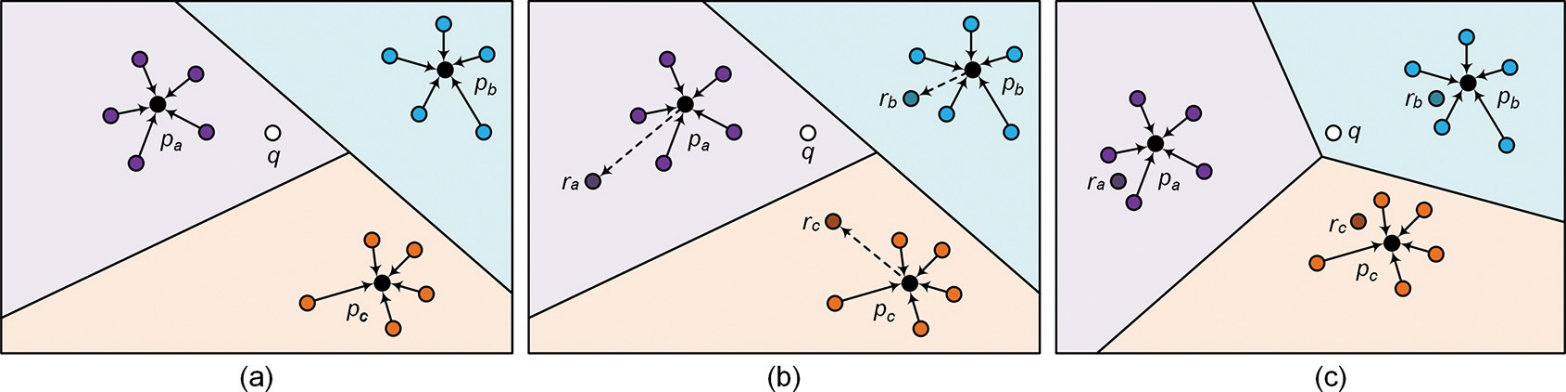
The illustration of knowledge enhancement mechanism. (a) Original prototype distribution, the query instance *q* closest to the prototype *p*^*a*^. (b) The distribution of the prototype has modified through prototype enhancement module, where *r* represent the relation information. (c) Updated prototype distribution, the query instance *q* is closest to the prototype *p*^*b*^, which is correctly classified now.

Prototypical networks perform classification by calculating the metric space distance between query instances and class prototypes. Typically, prototypes are derived from the average of supported instances, which lack reliable a priori knowledge. As depicted in Fig 2, external knowledge such as relation label and description can provide strong supporting evidence for the relation classification task, and this information is readily available for the FSRC task. Consequently, this paper employs relation label and description as supplementary data to enhance the prototype representation.

#### 4.2.1 Basic prototypes

Following the typical configuration of prototypical networks, the sentence encoder encodes *K* instances in *N* classes. For each relation, the mean of *K* instances are used as the basic prototype *P*_*bas*_, with the following calculation formula as follows:

$$P_{bas}^{n}=\frac{1}{K}\sum_{k=1}^{K}f_{\theta }(s_{k}^{n})$$ (1)

where *f*_*θ*_() represents the sentence encoder.

#### 4.2.2 Enhanced prototypes

**Fig 3:**
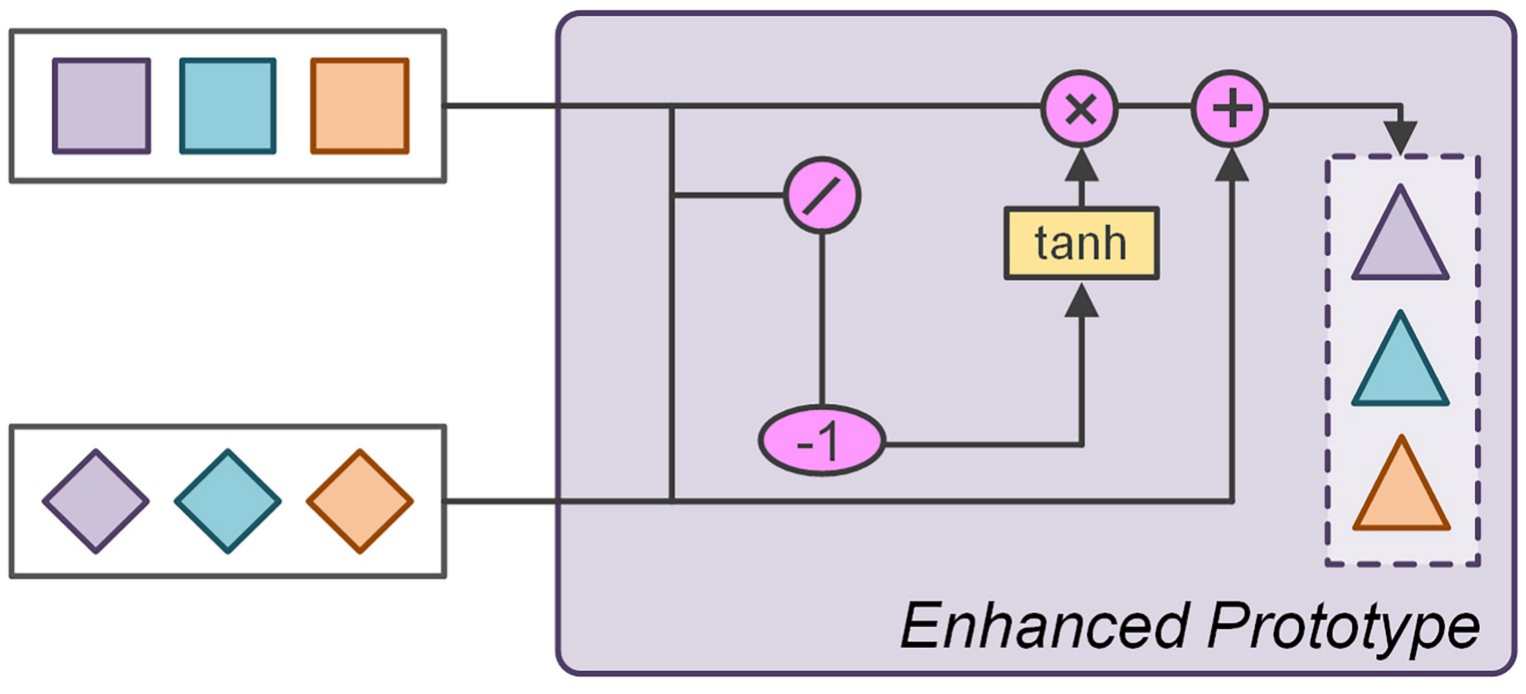
Structure of the prototype enhancement module. The rectangle, diamond, and triangle represent relationship information, basic prototype representation, and enhanced prototype representation, respectively.

To prevent the over-fitting problem caused by an excessive number of parameters, as depicted in Fig 3, we propose a non-weighted prototype enhanced module to combine the basic prototype with relational information. First, the global and local representations of the relation are concatenated as the final representation *R*_*rep*_ of the relational information as follows:

$$R_{rep}=R_{glo}⊕R_{loc}$$ (2)

The feature-level similarity between the relational information and the basic prototype is then determined through element-by-element division, and the similarity is mapped as the update signal of the gate *G* via the hyperbolic tangent function.


$$G=\tanh\left|\frac{R_{rep}}{P_{bas}}-1\right|$$ (3)

Finally, the basic prototype and relationship information are fused via the gate to generate the enhanced prototype representation *P*_*enh*_. The redundant information with a high degree of similarity will be filtered, and the information with a low degree of similarity will be combined through direct addition.

$$P_{enh}^{n}=R_{rep}^{n}*G+P_{bas}^{n}$$ (4)

where $n=1,\ldots ,N,P_{enh}^{n}\in R^{2d}$. All the representations used above belong to the ℝ^2*d*^.

### 4.3 Class cluster loss

**Fig 4:**
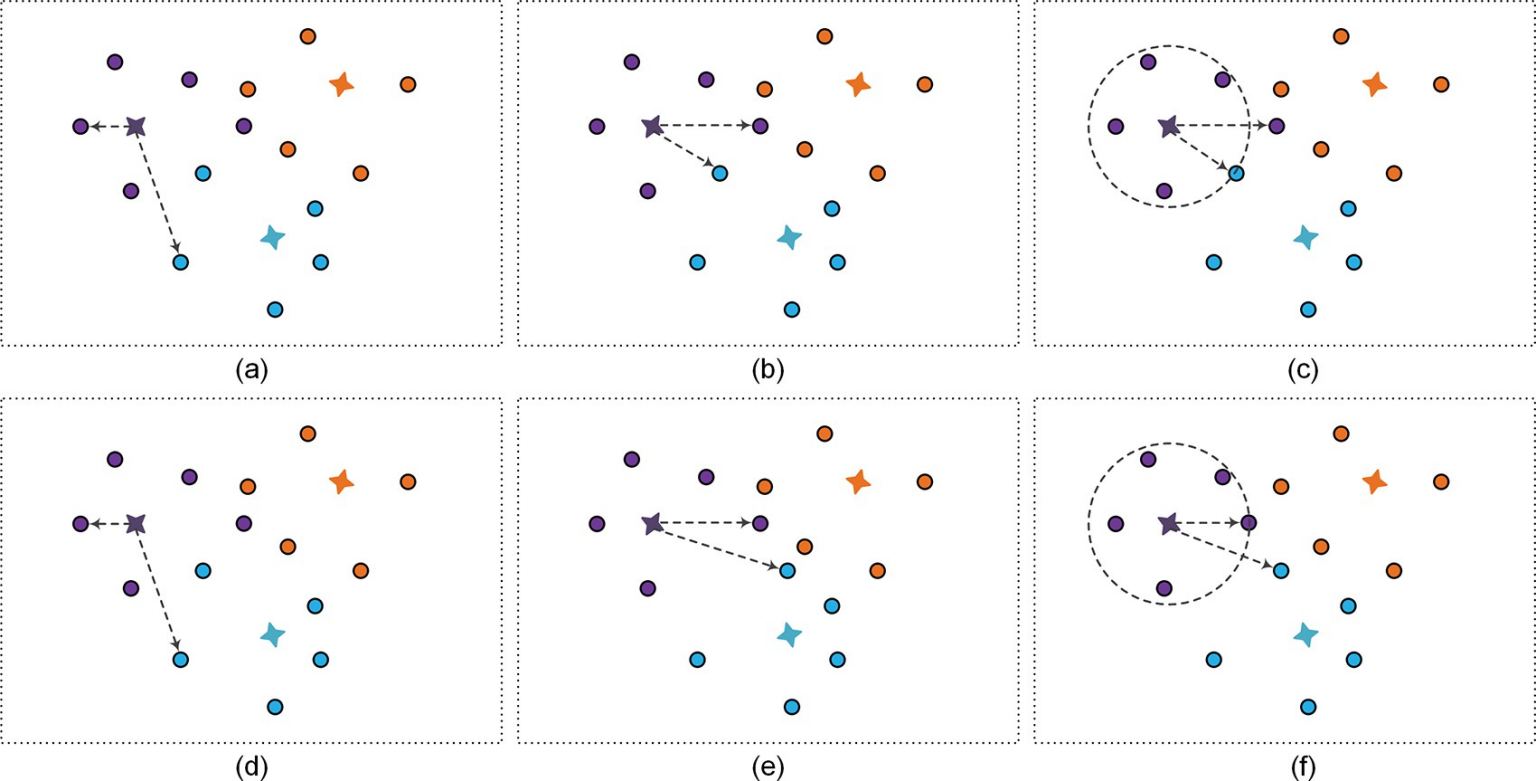
Illustration of class cluster loss. The (a), (b), and (c) represent the sampling strategies of triplet loss, triplet loss with hard sample mining, and class cluster loss, respectively. The (d), (e), and (f) respectively represent the updated metric space under the corresponding sampling strategy.

The performance of the FSRC model is greatly dependent on the distribution of instance vectors in metric space. To learn a metric space with high differentiation, a class cluster loss is designed. As illustrated in Fig 4, the primary objective of class cluster loss is to limit both intra-class compactness and inter-class separability.

#### 4.3.1 Triplet loss

Our work is improved based on triplet loss. The purpose of triplet loss is to distinguish different classes of samples, which can be defined as:

$$L_{tri}=relu(D^{a,p}-D^{a,n}+margin)$$ (5)

where *a*, *p* and *n* represent the anchor, positive and negative sample respectively. The *margin* is a hyperparameter to constrain the distance between dissimilar samples, and the *D* denotes the Euclidean distance between two samples.

#### 4.3.2 Hard sample mining

Random sampling can lead to a model that performs inconsistently and takes a long time to learn, so it’s best to think about hard sample mining. Following the work of Xiao et al. [12], we choose as optimization objects the farthest positive example and the closest negative example. The difference is that we chose the enhanced prototype as anchor, which makes the data distribution more stable. The hard triplet loss can be defined as:

$$L_{trih}=relu(D_{max}^{pro,p}-D_{min}^{pro,n}+margin)$$ (6)

#### 4.3.3. Class cluster loss

Triplet Loss only considers the margins of inter-class separability, which causes the model to be limited in processing outlier samples. Thus, we design a class cluster loss based on triplet loss. First, the central distance *c* is derived by mean the distances of similar samples to the prototype, as follows:

$$c=\frac{1}{K}\sum_{k=1}^{K}D^{a,p}$$ (7)

Then, in order to make the metric space more discriminatory, we imposed explicit constraints both on intra-class compactness and inter-class separability. The class cluster loss is defined as follows:

$$L_{cc}=relu(D_{max}^{pro,p}-D_{min}^{pro,n}+margin)+relu(D_{max}^{a,p}-c)$$ (8)

Finally, in order to find a superior classification plane, we combine cross-entropy loss and class cluster loss for co-training. The joint loss is defined as follows:

$$L_{joint}=-\log\left(z_{y}\right)+a*L_{cc}$$ (9)

where *z*_*y*_ represents the probability that the query instance belongs to relation class *y*, and the α is a hyperparameter to balance the loss function.

## 5 Experiments

In this section, we talk about the model comparison experiments, ablation studies, and visualization on the public FSRC dataset FewRel 1.0 [32] and FewRel 2.0 [33].

### 5.1 Dataset and evaluation

#### 5.1.1 Dataset

Our model is evaluated on the FewRel 1.0 and FewRel 2.0. FewRel 1.0 is a generic domain dataset, which is typically used for few-shot relation classification tasks. FewRel 1.0 utilizes Wikidata as a knowledge base in conjunction with distant supervision to identify datasets containing target relations from the Wikipedia news corpus. The final training, validation, and test sets contain 64, 16 and 20 relations, respectively, with 700 instances per relation. Each instance has an average of 24.99 tokens, for a total of 124,577 unique tokens. FewRel 2.0 is a more challenging dataset designed to assess the domain adaptation and "not of the above" recognition abilities of FSRC models. This dataset is composed of specialized biomedical knowledge, consisting of a total of 25 relations, each containing 100 instances. In this paper, we shall employ FewRel 2.0 to evaluate the domain generalization capabilities of the model.

#### 5.1.2 Evaluation

In accordance with FewRel settings, our model is trained based on the *N*−*way*−*K*−*shot*. The *N* and *K* respectively represent the number of relation classes and instances, and accuracy is used to evaluate the performance of the model as follow:

$$Accuracy=\frac{Y_{true}}{Y}$$ (10)

where *Y*_*true*_ and *Y* represent the number of correctly classified and the total number to be classified, respectively.

### 5.2 Comparable models

Our model is compared to twelve other models, including two CNN-based and ten BERT-based models. Specifically, the following models employ CNN as the sentence encoder: 1) **Proto-HATT** [25], a hybrid attention prototypical network, focuses on solving the noise problem. 2) **MLMAN** [7], an interactive prototype network through multi-level matching and aggregation. The following models use BERT as the sentence encoder: 3) **BERT-PAIR** [33], a model that pairs support instances with corresponding query instances and feeds them into BERT for relation prediction. 4) **Proto-BERT** [21], a network that classifies relations based on the query instance’s distance from the class prototype. 5) **REGRAB** [18], a Bayesian meta-learning method to generalize various prototypes more effectively. 6) **TD-Proto** [8], a method that utilizes a weighted gate mechanism to combine entity and relation description information in order to produce a knowledge-aware class prototype. 7) **CTEG** [34], a confusion-aware training method that employs Kullback-Leibler Divergence to improve the capacity to differentiate between true and confusion model relations. 8) **ConceptFERE** [26], a method that introduces entity concept information to provide strong supporting evidence for relation classification. 9) **HCRP** [9], a model that introduces relation label to contrastive learning prototype representations and focuses the model on difficult tasks by increasing the weight of difficult samples. 10) **MTB** [31], a pre-training model for learning relation representation from a large unsupervised corpus using entity-linked text based on Harris’ assumptions regarding distributional properties. 11) **CP** [35], a contrastive pre-training model. 12) **MapRE** [27], a relation mapping network that takes advantage of label knowledge.

### 5.3 Experiment setting

In Table 2, all hyperparameters are listed. As our experimental environment, we employed Transformer 4.7.0 and PyTorch 1.7.1 and trained on an RTX 3090Ti GPU. The pre-trained models BERT-base-uncased and CP are used, respectively, as sentence encoders in our model, where CP is a contrastive pre-training model based on BERT. The pre-training weights of the sentence encoder will be utilized as initialization parameters for fine-tuning on the FewRel dataset. The AdamW algorithm is utilized to solve the optimization problem, with the learning rates set to 1e-5 and 5e-6. We set the hidden layer to 768 and the batch size to 4. Iterations of training and validation are 20,000 and 4, respectively.

**Table 2:** Hyperparameter of the modes built in our experiments.

Component	Parameter	Value
**BERT**	type	base-uncased, CP
	hidden size	768
	max length	128
**Training**	learning rate	1e-5, 5e-6
	batch size	4
	max iterations	20,000
**Loss**	margin	0.5
	alpha	0.5

### 5.4 Result and discussion

#### 5.4.1 Comparison with different models

All the experimental results of FSRC models are shown in Tables 3 and 4. The evaluation results of the model on the generic domain dataset FewRel 1.0 are shown in Table 3, which includes CNN-based and BERT-based methods, and four conventional *N*−*way*−*K*−*shot* settings are adopted. Notably, the top half of the BERT-based models utilized the original BERT, while the bottom half models performed additional pre-training on the BERT. Among them, Proto-BERT is the basic model that does not introduce our proposed prototype enhancement module and class cluster loss. In this experiment, we apply our model to BERT and CP.

**Table 3:** Experimental results of different models on the FewRel 1.0 validation and test set.

Encoder	Model	5-w-1-s	5-w-5-s	10-w-1-s	10-w-5-s
**CNN**	Proto-HATT	72.65 / 74.52	86.15 / 88.40	60.13 / 62.38	76.20 / 80.45
	MLMAN	75.01 /—–—	87.09 / 90.12	62.48 /—–—	77.50 / 83.05
**BERT**	BERT-PAIR	85.66 / 88.32	89.48 / 93.22	76.84 / 80.63	81.76 / 87.02
	Proto-BERT	84.77 / 89.33	89.57 / 94.13	76.85 / 83.41	83.42 / 90.25
	REGRAB	87.95 / 90.30	92.54 / 94.25	80.26 / 84.09	86.72 / 89.93
	TD-Proto	—–—/ 84.76	—–—/ 92.38	—–—/ 74.32	—–—/ 85.92
	CTEG	84.72 / 88.11	92.52 / 95.25	76.01 / 81.29	84.89 / 91.33
	ConceptFERE	—–—/ 89.21	—–—/ 90.34	—–—/ 75.72	—–—/ 81.82
	HCRP	90.90 / 93.76	93.22 / 95.66	84.11 / 89.95	87.79 / 92.10
	**KEPN (ours)**	91.69 / **95.06**	93.74 / **96.44**	84.41 / **91.62**	87.53 / **93.71**
BERT*	MTB	—–—/ 91.10	—–—/ 95.40	—–—/ 84.30	—–—/ 91.80
	CP	—–—/ 95.10	—–—/ 97.10	—–—/ 91.20	—–—/ 94.70
	MapRE	—–—/ 95.73	—–—/ 97.84	—–—/ 93.18	—–—/ 95.64
	HCRP+CP	94.10 / 96.42	96.05 / 97.96	89.13 / 93.97	93.10 / 96.46
	**KEPN+CP**	96.57 / **97.51**	97.34 / **98.07**	93.72 / **95.44**	95.84 / **96.65**

The *N*−*w*−*K*−*s* denotes the task setting of *N*−*way*−*K*−*shot*. BETR* represents the BERT model with additional pre-training. These results can be found in original papers or CodaLab.

**Table 4:** Experimental results on FewRel 2.0 domain adaptation test set.

Model	5-w-1-s	5-w-5-s	10-w-1-s	10-w-5-s
Proto-CNN	35.09	49.37	22.98	35.22
Proto-BERT	40.12	51.50	26.45	36.93
BERT-PAIR	67.41	78.57	54.89	66.85
HCRP	76.34	83.03	63.77	72.94
MTB	74.77	87.91	62.53	81.17
CP	79.75	84.90	68.13	79.82
**KEPN (ours)**	80.26	89.14	69.61	83.78

Through Table 3, we can observe three results. First, KEPN reaches optimal accuracy on the test set. Second, our model achieves higher improvements on the tasks of 5−*way*−1−*shot* and 10−*way*−1−*shot*, which shows that KEPN is more compatible for few-shot tasks. Third, KEPN achieves a huge improvement compared to the basic model. These results demonstrate the effectiveness of our approach.

Our model achieved the best performance in the general domain. To further verify the model’s transferability, as shown in Table 4, we conducted tests on the biomedical dataset FewRel 2.0. It should be noted that in order to evaluate the domain generalization ability of the model, we only conducted training on the FewRel 1.0 dataset, and the FewRel 2.0 dataset was only used for testing. By observing Table 4, we can derive two results. Firstly, compared to the test results in the general domain, all models showed varying degrees of decline in performance. Secondly, KEPN outperformed other models in all tasks. These results demonstrate the generalization and effectiveness of our model and also highlight the importance of an enhanced prototype representation for FSRC tasks.

#### 5.4.2 Performance on different tasks

**Fig 5:**
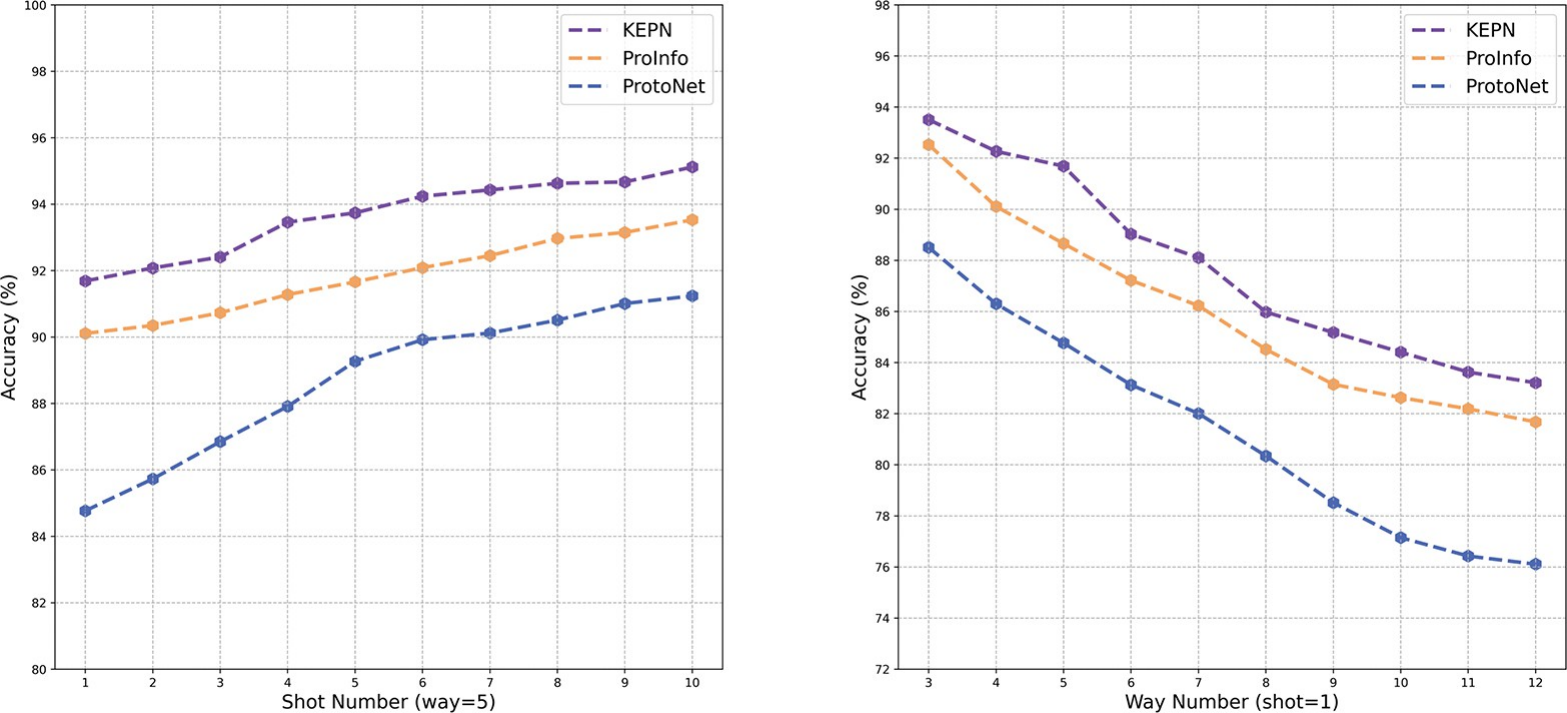
The performance on different tasks.

In order to verify the applicability of KEPN, we compare it with other models under different task settings on FewRel 1.0 validation set. First, K is changed from 1 to 10, when N to 5. Then, the range of N is 3–12 when K is fixed to 1. As shown in Fig 5, the model’s performance improves as the number of samples increases. It indicates that additional information can add more useful support clues to the prototype representation, which is consistent with our concept of utilizing relational information to improve the class prototype representation. The model’s accuracy degrades as the number of classes increases. This is expected because the model needs to account for more relational differences. Compared to the basic model, our model achieves greater accuracy, validating its robustness and generalizability.

#### 5.4.3 Statistical test

To further investigate whether there is a significant difference between the proposed method and the baseline model, we conducted a paired sample t-test [36]. Specifically, we initialized two models with identical random seeds and trained and validated them on the four “*N-way-K-shot*” tasks to obtain paired results. The training and validation iterations were set to 1000. This process was repeated seven times with different random seeds. Ultimately, we obtained seven pairs of test data. As shown in Table 5, the two-tailed P-values for both tasks were less than 0.05, indicating that our model significantly outperformed the baseline model.

**Table 5:** Paired-sample t-test on FewRel 1.0 validation set.

Task		5-w-1-s		5-w-5-s		10-w-1-s		10-w-5-s	
		ProtoNet	KEPN	ProtoNet	KEPN	ProtoNet	KEPN	ProtoNet	KEPN
**Seed**	25	84.63	88.71	88.18	91.01	77.94	81.02	81.92	83.31
	42	83.66	89.28	88.16	91.18	78.51	82.17	80.19	82.72
	77	84.32	88.21	88.96	90.65	78.89	81.09	81.51	83.71
	125	85.39	87.12	88.13	91.48	77.75	80.03	81.89	82.87
	633	84.09	88.22	87.95	90.98	79.80	81.01	81.79	84.13
	1059	84.29	89.39	87.79	91.73	78.66	82.72	80.87	82.66
	3407	85.96	87.73	88.16	91.46	78.43	80.62	82.33	84.83
**P-value**		7.6E-7		1.8E-4		2.1E-5		1.3E-4	

### 5.5 Ablation study

In Table 6, we discussed the impact of relation information, various fusion mechanisms, and loss functions on the prototype network’s performance. KEPN refers to the comprehensive model with the non-weighted prototype enhancement module and class cluster loss. The types "without relation info." and "with relation info." denote, respectively, the prototype network without introducing relation information and the various alternatives to the non-weighted fusion mechanism for relation information. The "w/ loss" indicates experimentation with alternative loss functions as opposed to class cluster loss.

**Table 6:** Ablation study on FewRel 1.0 validation set.

Model		5-w-1-s	10-w-1-s
KENP		91.69	84.41
**w/o relation info.**	local relation	89.04	80.29
	global relation	89.39	80.14
	relation info.	84.77	76.85
**w/ relation info.**	add	89.15	83.63
	concatenate	80.34	73.78
	weight gate	87.32	79.23
	hybrid attention	89.26	81.99
**w/ loss**	cross entropy	90.10	82.63
	triple loss	90.64	83.42

According to Table 6, three results can be observed. First, relation information is essential for prototype representation, and the model’s performance is enhanced most by the features that connect global and local relations. Second. the direct addition, concatenate, weighted gate, and hybrid attention perform poorly in terms of feature fusion. One possible explanation is that these methods introduce an excessive amount of redundant data or features, leading to an overfitting of the model. Thirdly, owing to our proposed constraints on both intra-class compactness and inter-class separability, our model achieves optimal performance relative to cross entropy loss and triplet loss. These results indicate that KEPN could more effectively represent prototypes and metric space.

### 5.6 Visualization

**Fig 6:**
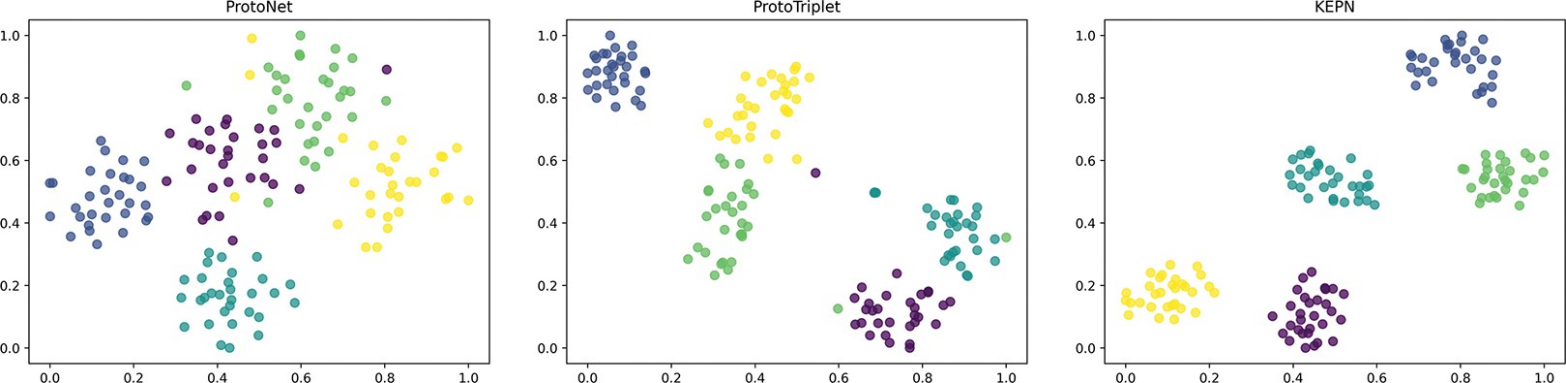
Visualization of instance embeddings of a 5-way-30-shot task on the FewRel 1.0 validation set.

To acquire a more intuitive grasp of KEPN, a collection of data was graphically represented. As depicted in Fig 6, the vanilla model labeled ProtoNet has a limited capacity to represent instance vectors. After introducing the triplet loss, the model named ProtoTriplet is able to distinguish between instances of various relational classes. Nevertheless, there are still outlier samples that are hard to classify, and the intraclass distance is not compact enough. The KEPN model with the prototype enhancement module and class cluster loss can learn a metric space with greater discriminability than the other model, enabling it to predict the relationships between entities more precisely. We believe that the few parameters and explicit constraints of the few-shot model are advantageous.

### 5.7 Case study

We displayed the classification results of several instances in the FewRel 1.0 validation set. Table 7 displays the five instances where the Proto-BERT classification was incorrect but our model classification was accurate. In the third instance of Table 7, Proto-BERT without prototype enhancement and class cluster loss incorrectly classifies the relation as "part of," whereas our model accurately predicts the relation as "member of." This is a challenging example because the semantic relationships of "part of" and "member of" are very similar. From Table 7, we could deduce that KEPN is capable of simulating the subtle distinctions between relations.

**Table 7:** Case study on FewRel 1.0 validation set.

Relation	Instance	Proto-BERT	CP	KEPN
**crosses**	The **Bridge of Dreams** is located on the *Mohican River* in Brinkhaven.	located in	crosses	crosses
**child**	She is the wife of Bollywood actor, **Jackie Shroff** and mother of *Tiger Shroff* and Krishna Shroff.	mother	child	child
**member of**	**Waylon** had previously represented the Netherlands as part of *The Common Linnets* alongside Ilse DeLange.	part of	member of	member of
**spouse**	He was born in Kristiania as a son of *Gerda Ring* and **Halfdan Christensen** and brother of Bab Christensen.	sibling	spouse	spouse
**military rank**	Rose is named after *Field marshal* **Hugh Rose**.	named after	named by	military rank

The text in bold and italics represents head and tail entities, respectively.

### 5.8 Hyperparameter study

**Fig 7:**
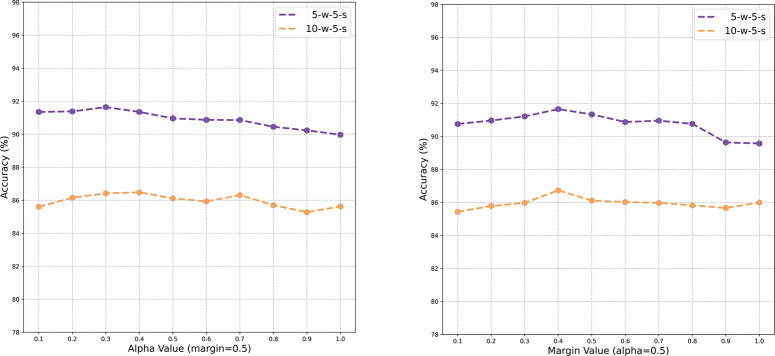
Hyperparameter study on FewRel 1.0 validation set.

In this section, we examined the impact of varying the numerical values of the hyperparameters margin and alpha on model performance. BERT was used as the pre-training model for this experiment, with 3000 and 1000 iterations for training and validation, respectively. Fig 7 shows the effects of different alpha and margin values on model performance for the 5-w-5-s and 10-w-5-s tasks. Firstly, when the margin was set to 0.5, alpha was increased from 0.1 to 1. Then, with alpha fixed at 0.5, the margin range was set to 0.1–1. We can observe that the model’s performance varies with changes in both parameter values, indicating that both alpha and margin affect the model’s performance. The best model performance is achieved when the margin and alpha values are close to 0.4. However, in terms of accuracy, the model is not very sensitive to the values of alpha and margin. This may be due to the fact that there are more simple tasks which cause cross-entropy loss to play a more important role in the early stages of training.

## 6 Conclusions and future work

This paper focuses on FSRC and proposes a knowledge-enhanced prototypical network. The central idea of our model is to utilize the feature-level similarity of the prototype and relational information to filter and fuse the information through a non-weighted gate mechanism, which enhances the prototype representation while avoiding the overfitting problem caused by too many parameters. In addition, we design a class cluster loss that optimizes those positive and negative samples that are difficult to classify during the training process and explicitly constrains both intra-class compactness and inter-class separability to learn a more discriminative metric space. As a result, KEPN attains the best accuracy on the FewRel 1.0. We believe that fewer parameters and explicit constraints are meaningful and can be generalized to other few-shot classification tasks. However, there are still several limitations that must be considered. Firstly, the model’s performance will be limited when applied to more complex datasets. Secondly, the model is dependent on the fixed relation classes during the learning process, which is disadvantageous for continuous learning. In future research, we will focus on developing more flexible and adaptable methods to handle a wider range of relation classification tasks, such as those in the biological or civil language processing domains. Additionally, introducing incremental learning into few-shot classification tasks may be necessary to address the continuous addition of new relation classes in real-world scenarios.

## Supporting information

S1 File Dataset and code. (DOCX)

## References

[1] Liu J, Duan X, Zhang R, Sun Y, Guan L, Lin B. Relation classification via BERT with piecewise convolution and focal loss. Plos one. 2021;16(9):1–23. doi: 10.1371/journal.pone.0257092 34506554 PMC8432804

[2] Wang L, Cao Z, De Melo G, Liu Z, editors. Relation classification via multi-level attention cnns. Proceedings of the 54th Annual Meeting of the Association for Computational Linguistics (Volume 1: Long Papers); 2016. p. 1298–1307.

[3] Mintz M, Bills S, Snow R, Jurafsky D, editors. Distant supervision for relation extraction without labeled data. Proceedings of the Joint Conference of the 47th Annual Meeting of the ACL and the 4th International Joint Conference on Natural Language Processing of the AFNLP; 2009. p. 1003–1011.

[4] Wang M, Cai Y, Gao L, Feng R, Jiao Q, Ma X, et al. Study on the evolution of Chinese characters based on few-shot learning: From oracle bone inscriptions to regular script. Plos one. 2022;17(8):1–17. doi: 10.1371/journal.pone.0272974 35984774 PMC9390942

[5] Zhang Y, Fang M, Wang N. Channel-spatial attention network for fewshot classification. Plos one. 2019;14(12):1–16. 10.1371/journal.pone.0225426 PMC6907821 31830065

[6] Sun S, Sun Q, Zhou K, Lv T, editors. Hierarchical attention prototypical networks for few-shot text classification. Proceedings of the 2019 conference on empirical methods in natural language processing and the 9th international joint conference on natural language processing (EMNLP-IJCNLP); 2019. p. 476–485.

[7] Ye Z-X, Ling Z-H, editors. Multi-Level Matching and Aggregation Network for Few-Shot Relation Classification. Proceedings of the 57th Annual Meeting of the Association for Computational Linguistics; 2019. p. 2872–2881.

[8] Yang K, Zheng N, Dai X, He L, Huang S, Chen J, editors. Enhance prototypical network with text descriptions for few-shot relation classification. Proceedings of the 29th ACM International Conference on Information & Knowledge Management; 2020. p. 2273–2276.

[9] Han J, Cheng B, Lu W, editors. Exploring Task Difficulty for Few-Shot Relation Extraction. Proceedings of the 2021 Conference on Empirical Methods in Natural Language Processing; 2021. p. 2605–2616.

[10] Lever J, Krzywinski M, Altman N. Points of significance: model selection and overfitting. Nature methods. 2016;13(9):703–5.

[11] Fan M, Bai Y, Sun M, Li P, editors. Large margin prototypical network for few-shot relation classification with fine-grained features. Proceedings of the 28th ACM International Conference on Information and Knowledge Management; 2019. p. 2353–2356.

[12] Xiao Y, Jin Y, Hao K. Adaptive prototypical networks with label words and joint representation learning for few-shot relation classification. IEEE Transactions on Neural Networks and Learning Systems. 2021. 10.1109/TNNLS.2021.3105377 34495842

[13] Chen W, Chen X, Zhang J, Huang K, editors. Beyond triplet loss: a deep quadruplet network for person re-identification. Proceedings of the IEEE conference on computer vision and pattern recognition; 2017.

[14] Finn C, Abbeel P, Levine S, editors. Model-agnostic meta-learning for fast adaptation of deep networks. International conference on machine learning; PMLR, 2017: 1126–1135.

[15] Nichol A, Achiam J, Schulman J. On first-order meta-learning algorithms. arXiv preprint arXiv:180302999. 2018.

[16] Ravi S, Larochelle H, editors. Optimization as a model for few-shot learning. International conference on learning representations; 2017.

[17] Dong B, Yao Y, Xie R, Gao T, Han X, Liu Z, et al., editors. Meta-information guided meta-learning for few-shot relation classification. Proceedings of the 28th international conference on computational linguistics; 2020. p. 1594–1605.

[18] Qu M, Gao T, Xhonneux L-P, Tang J, editors. Few-shot relation extraction via bayesian meta-learning on relation graphs. International conference on machine learning; 2020. p. 7867–7876.

[19] Koch G, Zemel R, Salakhutdinov R, editors. Siamese neural networks for one-shot image recognition. ICML deep learning workshop; 2015.

[20] Vinyals O, Blundell C, Lillicrap T, Wierstra D. Matching networks for one shot learning. Advances in neural information processing systems. 2016.

[21] Snell J, Swersky K, Zemel R. Prototypical networks for few-shot learning. Advances in neural information processing systems. 2017.

[22] Zhang D, Confidence R, Anazodo U, editors. Stroke Lesion Segmentation from Low-Quality and Few-Shot MRIs via Similarity-Weighted Self-ensembling Framework. Medical Image Computing and Computer Assisted Intervention–MICCAI 2022: 25th International Conference, Singapore, September 18–22, 2022, Proceedings, Part V. Cham: Springer Nature Switzerland, 2022: 87–96.

[23] Feng T, Qi Q, Wang J, Liao J, editors. Few-shot class-adaptive anomaly detection with model-agnostic meta-learning. 2021 IFIP Networking Conference (IFIP Networking); 2021: 1–9.

[24] Mozafari M, Farahbakhsh R, Crespi N. Cross-lingual few-shot hate speech and offensive language detection using meta learning. IEEE Access. 2022;10: 14880–14896.

[25] Gao T, Han X, Liu Z, Sun M, editors. Hybrid attention-based prototypical networks for noisy few-shot relation classification. Proceedings of the AAAI conference on artificial intelligence; 2019. p. 6407–6414.

[26] Yang S, Zhang Y, Niu G, Zhao Q, Pu S, editors. Entity Concept-enhanced Few-shot Relation Extraction. Proceedings of the 59th Annual Meeting of the Association for Computational Linguistics and the 11th International Joint Conference on Natural Language Processing (Volume 2: Short Papers); 2021. p.987-991.

[27] Dong M, Pan C, Luo Z, editors. MapRE: An Effective Semantic Mapping Approach for Low-resource Relation Extraction. Proceedings of the 2021 Conference on Empirical Methods in Natural Language Processing; 2021. p. 2694–2704.

[28] Liu J, Guo X, Yuan Y, editors. Prototypical interaction graph for unsupervised domain adaptation in surgical instrument segmentation. Medical Image Computing and Computer Assisted Intervention–MICCAI 2021: 24th International Conference, Strasbourg, France, September 27–October 1, 2021, Proceedings, Part III 24. Springer International Publishing, 2021: 272–281.

[29] Yarats D, Fergus R, Lazaric A, Pinto L, editors. Reinforcement learning with prototypical representations. International Conference on Machine Learning; PMLR, 2021: 11920–11931.

[30] Hospedales T, Antoniou A, Micaelli P, Storkey A. Meta-learning in neural networks: A survey. IEEE transactions on pattern analysis and machine intelligence. 2021;44(9):5149–69. 10.1109/TPAMI.2021.3079209 33974543

[31] Soares LB, Fitzgerald N, Ling J, Kwiatkowski T, editors. Matching the Blanks: Distributional Similarity for Relation Learning. Proceedings of the 57th Annual Meeting of the Association for Computational Linguistics; 2019. p. 2895–2905.

[32] Han X, Zhu H, Yu P, Wang Z, Yao Y, Liu Z, et al., editors. FewRel: A Large-Scale Supervised Few-Shot Relation Classification Dataset with State-of-the-Art Evaluation. Proceedings of the 2018 Conference on Empirical Methods in Natural Language Processing; 2018. p. 4803–4809.

[33] Gao T, Han X, Zhu H, Liu Z, Li P, Sun M, et al., editors. FewRel 2.0: Towards More Challenging Few-Shot Relation Classification. Proceedings of the 2019 Conference on Empirical Methods in Natural Language Processing and the 9th International Joint Conference on Natural Language Processing (EMNLP-IJCNLP); 2019. p.6250-6255.

[34] Wang Y, Bao J, Liu G, Wu Y, He X, Zhou B, et al., editors. Learning to Decouple Relations: Few-Shot Relation Classification with Entity-Guided Attention and Confusion-Aware Training. Proceedings of the 28th International Conference on Computational Linguistics; 2020. p. 5799–5809.

[35] Peng H, Gao T, Han X, Lin Y, Li P, Liu Z, et al., editors. Learning from Context or Names? An Empirical Study on Neural Relation Extraction. Proceedings of the 2020 Conference on Empirical Methods in Natural Language Processing (EMNLP); 2020. p. 3661–3672.

[36] Mee RW, Chua TC. Regression toward the mean and the paired sample t test. The American Statistician. 1991;45(1):39–42.

