# Supplementary material for: Knowledge-enhanced prototypical network with class cluster loss for few-shot relation classification
Source: PLoS One. 2023 Jun 8;18(6):e0286915. doi: 10.1371/journal.pone.0286915 (PMC10249838; doi:10.1371/journal.pone.0286915)
Supplement: S1 File — (DOCX) [file pone.0286915.s001.docx]

**Supporting information**

**Dataset**

The FewRel 1.0 dataset can be downloaded from the link:

<https://www.kaggle.com/datasets/taoliu25/fewrel-10>.

The FewRel 2.0 dataset can be downloaded from the link:

<https://www.kaggle.com/datasets/taoliu25/fewrel-20>.

**Code**

The source code is available at <https://github.com/Canopee25/KEPN>.

**Main results**

Main results can be found at CodaLab <https://codalab.lisn.upsaclay.fr/competitions/7395#results>.

**Backend models**

BERT: <https://huggingface.co/bert-base-uncased>.

CP: <https://github.com/thunlp/RE-Context-or-Names/tree/master/pretrain>.

**Trained models**

BERT: <https://drive.google.com/drive/folders/1qK_zKlbC6CbiTGsaLAkForfo-L3k1wpw?usp=share_link>.

CP: <https://drive.google.com/drive/folders/1aNoHYY6jVM_PkZD5xUqMfra8NSh5Rjyq?usp=share_link>.
